# Intersecting inequalities in experiences of violence among Brazilian adults: a multilevel analysis of individual heterogeneity and discriminatory accuracy (MAIHDA) of the 2019 National Health Survey

**DOI:** 10.1186/s12939-026-02818-x

**Published:** 2026-03-24

**Authors:** Wilson H. Hammett, James Macinko

**Affiliations:** 1 Department of Community Health Sciences, Fielding School of Public Health, University of California, Suite 36-071 CHS, Box 951772, Los Angeles, CA 90095-1772 USA; 2 Department of Health Policy and Management, Fielding School of Public Health, University of California, Los Angeles, USA

**Keywords:** Interpersonal violence, MAIHDA, Multilevel model, Intersectionality, Health equity

## Abstract

**Introduction:**

Existing quantitative studies of violence victimization in Brazil often examine individual demographic and socioeconomic risk factors, limiting insight into how identities can intersect to co-produce vulnerability or resilience. This study uses a nationally representative household survey to investigate how demographic, socioeconomic, and geographic factors intersect to shape the probability of experiencing psychological, physical, and sexual violence among Brazilian adults.

**Methods:**

Data from the 2019 Brazil National Health Survey was used to created indicators of 12-month experience of three types of interpersonal violence (psychological, physical, and sexual), a measure of any violence and one indicating 2 or more types. Previous literature guided the development of 356 clusters of intersectional identities based on demographic, socioeconomic and other factors. Analyses used the intersectional multilevel analysis of individual heterogeneity and discriminatory accuracy (MAIHDA) approach based on multilevel analyses of all 356 intersectional strata in addition to individual-level factors.

**Results:**

Among Brazilian adults, 18.3% (totaling 27,535,272) reported experiencing interpersonal violence and 3.7% experienced more than one type in the past 12 months. Psychological violence (17.4%) was most frequently reported, followed by physical (4.6%) and sexual (0.8%) violence. MAIHDA models revealed that prevalence and risk varied widely across intersectional strata, but that younger age (< 30), being single, living in an urban area, and living with a long-term illness or disability were consistently found in the strata with highest predicted probability of victimization across all types of violence. Being female, being Black, having a college-level education, and being in the lowest wealth tertile were also commonly found in the highest ranked strata across forms of violence victimization. The overall variance attributable to intersectional (as opposed to individual) effects was between 9.3% and 13.0% across different forms of violence, suggesting that risk of experiencing (or reporting) interpersonal violence in this study accumulates largely in additive rather than multiplicative ways.

**Conclusions:**

This study found that experiences of psychological, physical, and sexual interpersonal violence were patterned by intersecting social and economic inequalities, with higher risk among women, younger adults, Black or Brown individuals, those who are single, urban residents, and people living with long-term health problems. MAIHDA analyses revealed that risk accumulated across overlapping social positions—particularly among young, single, urban Black women with chronic conditions—highlighting the need for violence prevention strategies that address structural drivers of gender, racial, and socioeconomic inequality.

**Clinical trial number:**

Not applicable.

**Supplementary Information:**

The online version contains supplementary material available at 10.1186/s12939-026-02818-x.

## Introduction

Despite recent declines, interpersonal violence is prevalent in Brazil. In 2023, 45,747 people died due to homicide in Brazil (19.3 deaths/100,000), which represents a 20% decline from a decade earlier, but was still one of the highest rates observed in the world [1]. Self-reported experience of interpersonal violence is similarly prevalent, with almost one in five adults (18%) surveyed in 2019 reporting experiencing some type of interpersonal violence (psychological, physical, or sexual) in the last year [2]. Beyond possible physical injury, experiencing interpersonal violence has been shown to be strongly associated with mental health outcomes like depression, anxiety, and PTSD [3, 4], and can lead to increased risk of cardiovascular disease and premature mortality [4]. 

In this article, we define interpersonal violence—which may be physical, psychological, or sexual in nature—as violence against individuals or groups that takes place between family members and/or intimate partners or between acquaintances or strangers [5, 6]. We exclude examination of self-directed violence, war and state-sponsored, and structural violence. It is nevertheless important to acknowledge that any of these types of violence may co-occur along with interpersonal violence. Particularly important is the relationship between structural violence as a system that creates and maintains inequalities in power and resources and which can be thought of as a fundamental cause of interpersonal violence [7]. 

Johan Galtung’s theory of structural violence posits that structural factors—such as the political, legal, economic, and patriarchal institutional systems in which we live and work—manufacture deliberate systemic inequalities and that cause and perpetuate harm to marginalized communities [8, 9]. Entrenched in daily life, structural violence that systematically privileges certain identities over others is the “invisible” underlying cause of poor health outcomes [10, 11] and direct violence, or violence that can be seen, such as verbal or physical abuse [12]. Indeed, a growing body of literature has documented how factors such as gender, race, socioeconomic status, educational attainment, and place of residence further stratify risk of and protection from experiencing interpersonal violence in Brazil [13–15]. In particular, race and gender are marginalized identities that can demonstrate how shared experiences and structural factors of marginalization may shape risk of experiencing interpersonal violence [16]. For instance, Brazilian women report higher rates of experiencing physical violence than men, and the association between violence victimization and depression is stronger among women than men [2, 17]. Young adults (ages 18–29), and those who identify as Black or mixed race are also more likely to report experiencing violence [2]. Black and Brown women in Brazil have been found to report higher rates of self-reported violence victimization than white women, suggesting that exposure to racism and sexism may jointly shape vulnerability in complex ways [18]. 

Other individual-level factors that reflect structural inequities have also been shown to shape patterns of interpersonal violence victimization in Brazil, such as education level- a well-known fundamental cause of health inequities [13], relationship status- whereby those who are single may be at greater risk [13, 19, 20], urbanicity- due to its association in Brazil with unstandardized housing and persistent neighborhood deprivation [20, 21], and income level – given the flexible resources it provides [13]. 

Recent studies have examined many aspects of violence at the population level in Brazil. These include studies of the overall prevalence of violent experiences [2, 13], prevalence of certain types of violence such as intimate partner violence [14] or workplace psychological violence or harassment [22], as well as cross-sectional analyses examining the relationship between factors such as depression [23], sexual orientation and gender identity [24], and self-rated health and experiences of violence [25]. However, despite the widespread use of population-level datasets, most quantitative studies to date have examined single axes of risk or inequality and there has yet to be an analysis of the way that intersecting factors and identities may increase risk of or protect individuals from experiencing violence.

Existing quantitative examinations of violence victimization in Brazil often examine risk factors in isolation, limiting insight into how intersecting demographic and socioeconomic identities and structural conditions can co-produce vulnerability or resilience. However, intersectionality provides a key theoretical framework for examining patterns of health inequality because it emphasizes the multidimensional and interdependent nature of vulnerability [26–28]. Originally developed from Black feminist scholarship [28, 29], intersectional theory posits that social categories like gender, race, and class interlock to generate distinctive patterns of vulnerability and resilience, rather than acting independently [26–28, 30]. 

Drawing on this intersectional theory, considering the effects of multiple marginalized identities—such as being Black, being a woman, or being of low socioeconomic status—on health outcomes additively (i.e., simply through the main effects of regression analysis) would not adequately capture the heightened effects of holding multiple marginalized identities. The promise of intersectional quantitative analysis is to quantify multiplicative effects that more adequately describe the increased risks among people who experience more than one marginalized identity. Quantitively applying intersectional approaches to understanding experiences of violence aims to move beyond additive models and to explicitly assess and quantify how such overlapping social positions may further amplify or mitigate risk across a range of intersectional profiles [31]. 

This study uses a nationally representative household survey from Brazil to investigate how intersecting demographic and socioeconomic positions shape the probability of experiencing different forms of violence among Brazilian adults, helping to explain patterns of risk and protection that have not been captured through single factor analyses. We hypothesized that multiple vulnerabilities would contribute to reported experiences of violence, especially among women and people of color. Our findings aim to contribute empirical evidence on the multidimensional social determinants of violence in Brazil and to inform policies and interventions tailored to the most vulnerable intersections of risk factors.

## Methods

### Data and measures

We used data from the 2019 Brazil National Health Survey (*Pesquisa Nacional de Saúde* -PNS) [32], conducted by the Brazilian Census Bureau and Ministry of Health and with a sample size of 83,942 adults. The PNS uses a complex sample design in three stages and adult respondents are randomly selected from the households for more detailed interviews. Data collection occurs via pre-scheduled face-to-face interviews using structured questionnaires that cover a wide range of demographic, socioeconomic, and health-related questions [33]. 

The PNS asks individuals a series of questions about the types of violence they may have experienced, their frequency, and information about location of the event as well as the perpetrator. Prior to initiating the violence module (asked of all adults), the interviewer must indicate that the interview was conducted in a safe and private location. We created binary indicators of three main types of violence: psychological violence (any form of in-person or online humiliation, harassment, or threat), physical violence (based on affirmative response to any of 5 questions), and sexual violence (based on 4 questions) (See appendix Table 3). The recall period for all measures is the past 12 months, except for two items on sexual violence for which the recall period is one’s lifetime, presumably due to their much lower prevalence rates. We also constructed a measure of any violence, which was coded as positive if the individual responded affirmatively to any of the previous violence questions and a final measure identifying individuals experiencing more than one type of violence.

Based on previous literature [13, 25, 34], we constructed a series of variables suitable for intersectional analyses. These include: respondent age (divided into categories of 18–29 and 30+), birth sex (male, female), race/skin color (Black, Brown or *parda*, white, all others), relationship status (single versus partnered), education (less than primary school, primary complete, high school complete, and any college or more), tertiles of household assets (derived from principal components analysis of a list of household goods), quintiles of household income, poor/fair self-rated health, and geography (rural versus urban, capital city versus rest of state, and the 5 Brazilian macro-regions). We also include a binary measure of any long-standing illness or disability from a list of doctor-diagnosed conditions and any long-term moderate or severe movement, vision, hearing or intellectual disability given their association with higher risk of experiencing interpersonal violence [35]. We included only those conditions that occurred more than one year ago, prior to the report of violence (in the past 12 months) and excluded all mental health diagnoses due to their strong association with violence [36] to avoid potentially unclear temporal ordering.

### Statistical analysis

In descriptive and bivariate analyses, differences in survey-adjusted and weighted proportions were compared using Chi-squared tests with a Rao-Scott adjustment [37]. For intersectionality analyses, we employed the intersectional multilevel analysis of individual heterogeneity and discriminatory accuracy (MAIHDA) approach [38, 39]. This technique makes use of multilevel models to create a series of clusters or strata of all possible intersectional identities, through high-dimensional, multi-categorical cross-tabulation. This method is particularly helpful in that it allows for examination of many intersecting factors (more so than through traditional interaction analyses) and further allows for a decomposition of the proportion of the observed outcome that can be attributed to intersectional (multiplicative) versus individual-level (additive) factors [40]. 

MAIHDA models were estimated separately for any and more than one type of interpersonal violence, and each violence sub-type to assess commonalities across types. Factors used to create the strata or clusters of intersectional identities were derived from the literature and tested empirically through logistic regression analyses. All socio-demographic and geographic factors that were statistically significantly associated with violence reports (*p* < 0.05) were retained for the intersectionality analysis, unless there were numerous strata with cell sizes of 10 or fewer. Based on these descriptive findings (See appendix Table 1), variables were simplified to represent the following groups: birth sex (male/female), age group (18–29 versus all others), race/skin color (Black versus all others), relationship status (single versus partnered), educational attainment (college versus others), household asset tertiles, rurality, and the presence of one or more long-standing chronic conditions were used for all analyses.

**Table 1:** Multilevel regression analysis, by outcome (*N* = 83,942)

Model	Any violence *N* = 14,602		Psychological violence *N* = 13,841		Physical violence *N* = 3,279		Sexual violence *N* = 641		2 + types of violence *N* = 2909	
	Null	Full	Null	Full	Null	Full	Null	Full	Null	Full
Male (v female)		0.89***		0.87***		1.03		0.46***		0.88*
		0.83,0.95		0.81,0.93		0.92,1.16		0.37,0.58		0.78,0.99
Age 18–29 (v 30+)		1.89***		1.85***		2.26***		2.66***		2.41***
		1.75,2.03		1.72,2.00		1.99,2.55		2.15,3.29		2.12,2.74
Black (v all others)		1.17***		1.15***		1.16*		1.22		1.16*
		1.1081.25		1.06,1.25		1.02,1.33		0.94,1.58		1.01,1.34
College+ (v less)		1.27***		1.27***		0.99		1.4***		1.05
		1.17,1.37		1.18,1.38		0.86,1.13		1.11,1.78		0.91,1.21
Assets 2 (v lowest)		0.97		0.99		0.78***		0.85		0.8**
		0.90,1.06		0.91,1.07		0.68,0.90		0.67,1.09		0.70,0.93
Assets 3 (highest)		0.79***		0.8***		0.56***		0.62**		0.57***
		0.72,0.86		0.73,0.88		0.48,0.66		0.47,0.83		0.48,0.67
Single (v partnered)		1.36***		1.34***		1.63***		2.32***		1.74***
		1.27,1.46		1.25,1.44		1.45,1.85		1.83,2.96		1.53,1.98
Any long-standing		1.4***		1.41***		1.41***		1.39***		1.49***
condition (v none)		1.30,1.50		1.31,1.51		1.26,1.59		1.13,1.72		1.32,1.68
Rural (v urban)		0.7***		0.7***		0.62***		0.82		0.63***
		0.64,0.76		0.65,0.77		0.53,0.72		0.63,1.08		0.54,0.74
Random effects	1.29***	1.03**	1.28***	1.03**	1.55***	1.06***	2.26***	1.08	0.04***	1.06***
Stratum-level	1.22,1.37	1.02,1.04	1.21,1.36	1.02,1.04	1.37,1.74	1.03,1.09	1.66,3.06	0.99,1.17	0.03,0.04	1.03,1.10
Variance partition coefficient (VPC)	7.20%	0.86%	7.02%	0.84%	11.69%	1.69%	19.83%	2.25%	13.1%	1.75%
Proportional Change in Variance (PCV)	-	88.8	-	88.8	-	87.0	-	90.7		88.1

Subsamples were not used for multivariable calculations, only for reporting selected prevalence rates for subgroups in descriptive statistics. Missing data in the PNS dataset is minimal, and there was no missing data for any outcome. For the full models, 25 respondents were dropped for having one or more missing covariates, resulting in a 0.03% missing variable rate. These response rates are consistent with those reported in other studies using the same dataset [2, 22]. 

## Results

Prevalence of reported 12-month experiences of any type of interpersonal violence among Brazilian non-institutionalized adults in 2019 was 18.27%, which represents over 25 million adults. Overall prevalence was highest for psychological violence (17.37%), followed by physical violence (4.6%), and sexual violence (0.78%). About 3.7% of respondents experienced more than one type of violence. See Appendix Fig. 1.

**Fig. 1:**
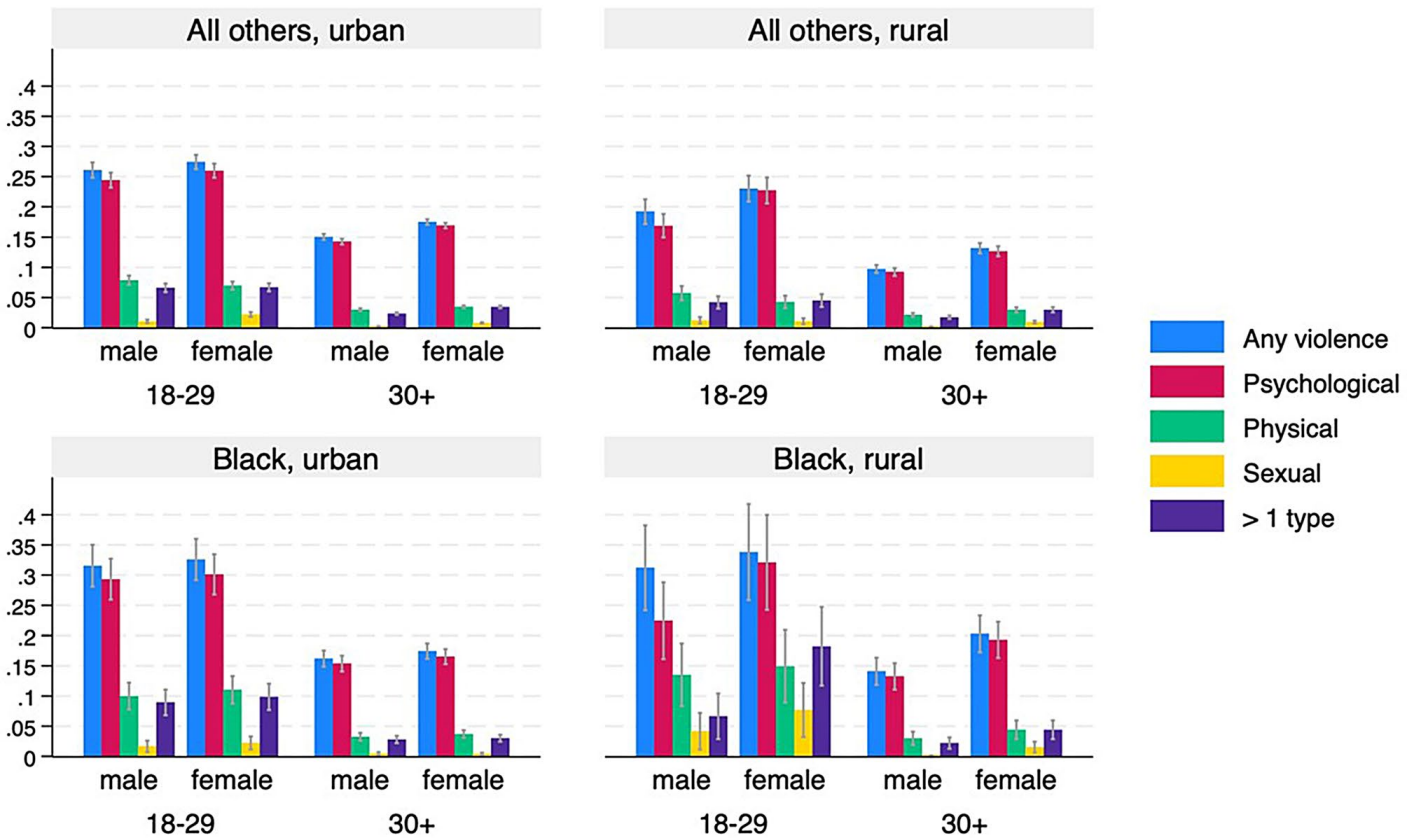
Prevalence of self-reported violence, by type, age, sex, race, and urbanicity. Bars represent weighted means with 95% confidence intervals

Figure 1 presents unadjusted survey-weighted means for four different intersectoral combinations. For each measure of interpersonal violence, prevalence rates are generally higher among women than for men (except for physical violence, *p* = 0.371), higher for younger than older populations, higher among Black individuals than other race/ethnicities (except in the case of sexual violence, *p* = 0.336), and higher in urban areas, but only for other race/ethnicities. The complexity of combinations of these four variables, the need for multiple hypothesis testing to compare different combinations, and the differing sample sizes (indicated by the differing confidence intervals) further motivates the use of the intersectional MAIHDA approach to examine a greater number of intersectional combinations.

Table 1 presents results from the MAIHDA models, which resulted in 356 strata based on all possible combinations of our eight stratifying variables (gender, age, race, educational attainment, household wealth tertiles, relationship status, illness/disability status, and urbanicity). For ease of presentation, we discuss results for each outcome in turn.

### Any interpersonal violence

In multilevel analyses (Table 1), lower odds of reporting experiencing any violence in the last 12 months (Adjusted Odds Ratio, AOR < 1) were statistically significantly associated with being male, being in the highest asset tertile, and living in a rural area. Higher odds (AOR > 1) were statistically significantly associated with younger age (18–29), being Black, having a college degree or higher, being single, and living with any long-term illness or disability. Notably, respondents in the highest education attainment category (undergraduate degree or higher) exhibited elevated odds of reporting any type of violence.

**Fig. 2:**
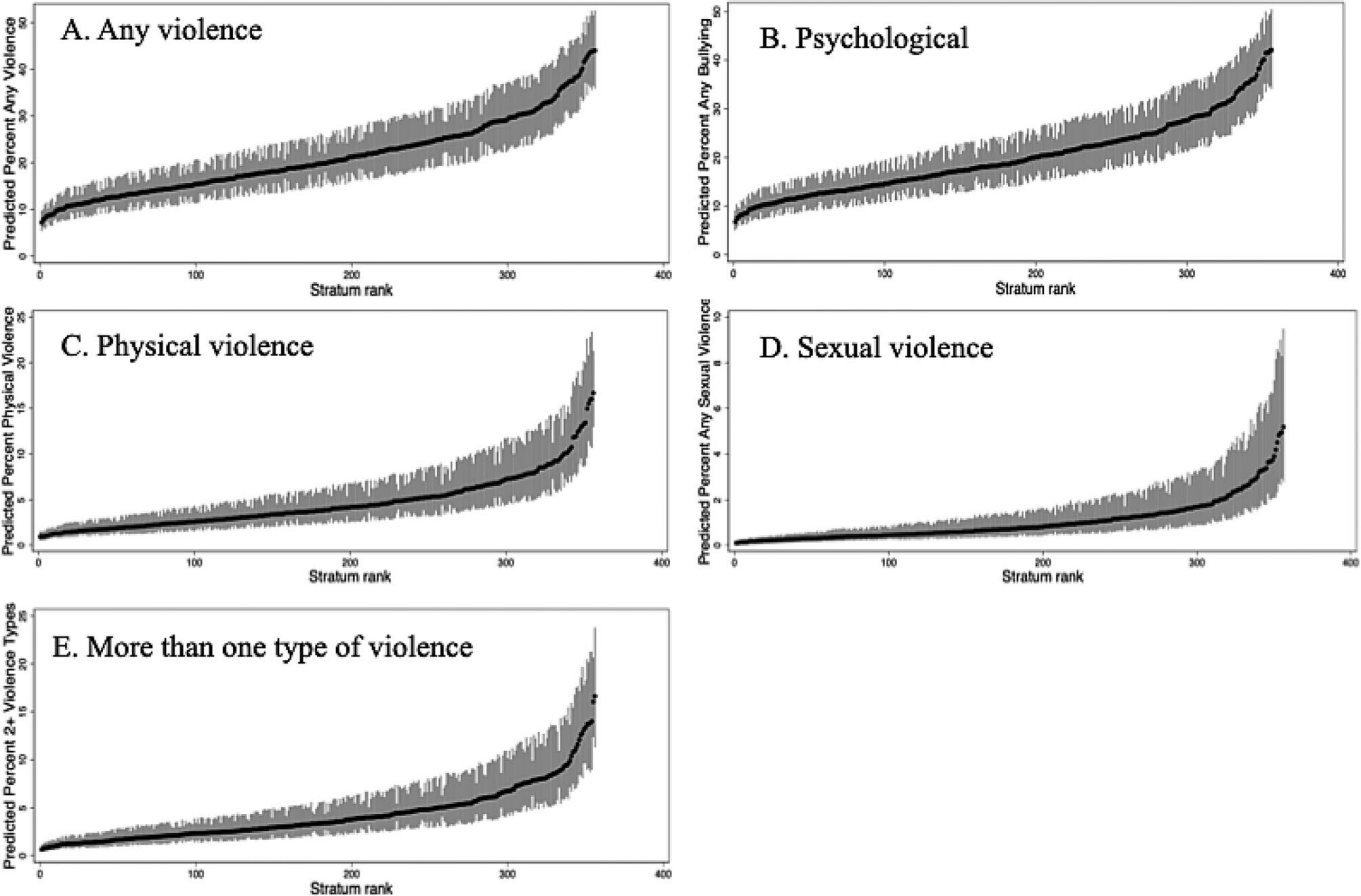
Predicted values by stratum for all strata, by outcome. Figures indicate the range of predicted probabilities, for each stratum, of reporting each outcome. The dark markers indicate predicted values for each stratum and the lighter-colored spikes indicate each stratum’s approximate 95% Confidence Intervals. Note differences in Y axes for each panel

MAIHDA results reveal substantial variation across strata in predicted 12-month probability of any violence victimization, ranging from 8% to 44% (Fig. 2, panel A). The lowest ranked stratum comprised of older (30+), partnered, Black men in the highest asset group, living in rural areas, and without long-term illness or disability but with less than college-level education attainment (predicted probability = 8.00%). In contrast, the highest ranked stratum included young (18–29), single, Black women in the lowest assets group and living in urban areas with any long-term illness or disability but with a college degree or higher (predicted probability = 44.05%) (Table 2).

**Table 2:**
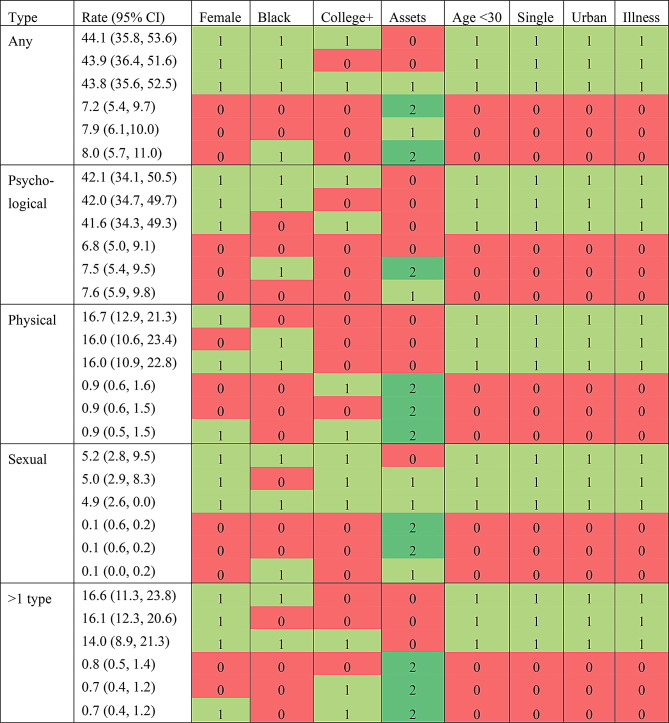
Intersections with the three highest and three lowest predicted prevalences, by outcome

Rate is the predicted probability of the outcome for that intersectional stratum and its 95% confidence interval

For all variables except assets, 0 = absent; 1 = present. For assets, 0 = low, 1 = middle, 2 = high

**Fig. 3:**
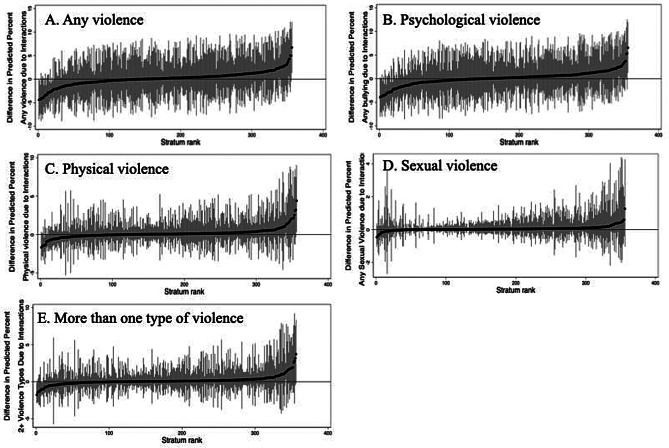
Predicted stratum interaction effects, by outcome. Figures indicate the difference in predicted percent of each outcome due to interactions (strata) for each outcome measure. The dark markers indicate predicted values for each stratum and the lighter-colored spikes indicate each stratum’s approximate 95% Confidence Intervals. Note differences in Y axes for each panel

Despite wide variation between predicted prevalence of different strata, statistics from Table 1 show the VPC (the proportion of total variance existing between strata), decreases from 7.2% in the null model to 0.86% in the full model, while the PCV in the full model is 88.8%, suggesting 11.2% of the variability in violence exposure lies within rather than between intersectional groups (Fig. 3, panel A).

### Psychological violence

Patterns for reported experience of 12-month psychological violence mirrored those for any violence. High education attainment (college degree or higher) was found to be associated with increased odds of reported experience of psychological violence (Table 1).

Predicted probability of 12-month experience of psychological violence ranged from 8% to 42%, following a pattern like that observed for any type of interpersonal violence (Fig. 2, Panel B). The lowest ranked stratum comprised of older (30+), partnered men in the “other” race category with less than college level education attainment, in the highest assets category, and living in rural areas with no long-term illness or disability (predicted probability = 7.64%), while the highest ranked stratum consisted of young (18–29) Black single women with a college degree or higher, in the lowest assets category, and living in urban areas with long-term illness or disability (predicted probability = 42.08%) (Table 2). As with any interpersonal violence, between-strata variance attributable to intersectional effects (100-PCV) for psychological violence was 11.2% and varied across strata (Fig. 3, panel B).

### Physical violence

For 12-month experience of physical violence, lower odds (AOR < 1) of reported victimization were statistically significantly associated with being in the middle or highest asset tertiles and living in a rural area (Table 1). Higher odds (AOR > 1) were statistically significantly associated with younger age (18–29), being Black, being single, and living with a long-term illness or disability (Table 1). Unlike other types of interpersonal violence, lower odds of reported physical victimization was not statistically significantly associated with being male.

Predicted probability of 12-month experience of physical violence ranged from 1% to 17%, indicating narrower variation across strata than for any violence or psychological violence (Fig. 2, panel C). The composition of the highest ranked strata differed from other outcomes, with female gender appearing in the lowest stratum and “other race” appearing in the highest stratum. However, characteristics in the lowest ranking strata (predicted probability = 0.94%) and highest-ranking strata (predicted probability = 16.69%) were otherwise found to be similar to those found for any violence and psychological violence (Table 2). Between-strata variation attributable to intersectional effects (100-PCV) was slightly higher than for other types of violence at 13.0%, with wide variation (Fig. 3, Panel C).

### Sexual violence

When reported experience of sexual violence was examined, fewer characteristics appeared to be protective, likely due to the small sample size (*N* = 641). Lower odds of reporting experiencing sexual violence in the last 12 months (AOR < 1) were found to be statistically significantly associated with being male and being in the highest asset tertile (Table 1). However, higher odds (AOR > 1) were found to be statistically significantly associated with younger age (18–29), higher education attainment (college degree or higher), being single, and living with any long-term illness or disability.

Predicted probability of experience of sexual violence was lowest among all violence types and showed the narrowest range (0%-5%) (Fig. 2, Panel D). The lowest ranked stratum included older (30+), partnered, Black men with less than college level education attainment in the middle assets category, and living in rural areas with no long-term illness or disability (predicted probability = 0.11%). The highest ranked stratum mirrored psychological violence and any violence, including younger (18–29), Black, single women in the lowest asset category but with a college degree or higher, living in urban areas with any long-term illness or disability (predicted probability = 5.19%) (Table 2). The total variance explained by intersectional strata (100-PCV) was the lowest of all other forms of violence at 9.3% and varied less across strata (Fig. 3, Panel D).

### More than one type of interpersonal violence

Factors associated with reporting more than one type of interpersonal violence included being female, younger age, black, lower household assets, being single, urban, and having any long-term illness or disability. There was no statistically significant association with educational attainment (Table 1).

The predicted probability of reporting more than one type of interpersonal violence ranged from 16.2% to less than 1% (Fig. 2, panel E). Black, younger women with less than college education, low assets, who are single, living in urban areas and have a long-term illness or disability showed the highest probability of more than one type of interpersonal violence (16%). Men who were “other race”, less than college education, high assets, aged 30 or over, partnered, living in rural areas and with no long-term illness or disability reported the lowest rates (less than 1%) (Table 2). The total variance explained by intersectional strata (100-PCV) was 11.9% and varied across strata (Fig. 3, Panel E).

## Discussion

This study examined how intersecting demographic and socioeconomic characteristics shape the likelihood of experiencing interpersonal violence among adults in Brazil, using nationally representative data from the 2019 National Health Survey (PNS). Prevalence of reported experience of interpersonal violence was found to support results of prior studies of the PNS 2019 (psychological = 17.36%, physical = 4.15%, and sexual = 0.76%) [13] and consistent with prior research, we found that experiences of violence were not evenly distributed across the population [13, 14]. Women, younger adults, and individuals identifying as Black or Brown were more likely to report experiencing violence than men, older adults, and white respondents. We also identified several additional factors that contribute to experiencing different forms of violence. These include adults who are single (rather than partnered), those who live in urban rather than rural areas, and individuals who have any long-term illness or disability were consistently found to be more likely to experience all types of violence than their peers.

Beyond identifying individual risk factors, we used a multilevel analysis of individual heterogeneity and discriminatory accuracy (MAIHDA) to explore how these characteristics combine to produce distinctive intersectional patterns associated with report of interpersonal violence. MAIHDA not only facilitates the examination of many intersectional strata at once, but also provides the ability to treat each stratum as the result of social and structural influences, rather than of inherent individual characteristics. These multiplicative, intersectional analyses provide a more nuanced view of how factors may combine to shape vulnerability across different demographic, socioeconomic, health, and geographic characteristics.

Our findings reinforce prior evidence that gender, race, wealth and geographic inequalities remain central to understanding risk of violent victimization in Brazil. Younger age (< 30), being single, living in an urban area, and living with a long-term illness or disability, were found in the strata with highest predicted probability of victimization across types of violence, while being female, being Black, having a college-level education, and being in the lowest wealth tertile were mostly, but not consistently, found in the highest ranked strata. Higher odds of reported violence among younger women, particularly psychological and sexual violence, align with established patterns of gendered rates of violence victimization in Brazil [2]. Elevated prevalence among Black and Brown respondents also reinforces existing scholarship documenting the racialization of violence exposure among Afro-Brazilian communities [2, 18]. These findings reinforce the theory that structural factors that create and maintain inequalities shape violence victimization, rather than simply the actions of individual perpetrators of interpersonal violence [8, 9]. These findings thus underscore the need for violence prevention interventions that recognize and address enduring racial and gender inequities as well as socioeconomic hierarchies [9, 12]. Similar applications of MAIHDA used in other contexts such as the United States [41] and England and Wales [42] have found that individuals possessing multiple marginalized identities show elevated prevalence of interpersonal violence victimization.

Experiencing any long-term illness or disability was consistently found in strata with the highest predicted probability, across all types of violence. Having a long-term illness or disability appears to be an independent predictor of experiencing violence, but this finding should be interpreted with some caution. Living with a long standing illness or disability could also be a consequence of experiencing violence (such as depression [43]) or of age (such as hypertension [44] or cancer [45, 46]). While 41% of the sample reported experiencing at least one long-standing condition or disability, only 18% of those under age 30 reported living with a chronic condition. Interestingly, the relationship between age, long-term illness/disability and reporting of interpersonal violence is complex, with a statistically significant interaction between illness and age such as that younger individuals with no long-term illness or disability had a 25.1% change of reporting violence, but those with any long-term health problem had a 35.6% prevalence of violence (p-value for interaction = 0.000). No such interaction was found among older populations. One explanation for this relationship is that, consistent with the literature, individuals living with a long-term illness or disability may be targeted by perpetrators of violence. Another, complementary explanation is that individuals who have experienced violence have been found to be more likely to use primary healthcare after victimization, which may provide opportunities for diagnosis of chronic conditions that would not otherwise have been diagnosed [47]. In prior analysis of the PNS 2019, any experience of interpersonal violence was found to be associated with higher odds of experiencing depression, although we did not include that condition on our list due to its strong association with violence and the possibility of reverse causation [23]. The cross-sectional nature of this study prevented us from determining whether certain conditions may be a result of violent experiences experienced earlier in life, consistent with the emerging literature on Adverse Childhood Experiences (or ACEs) [48, 49]. Future studies of the relationship between violence victimization and long-term illness should consider disaggregating by type of condition to better understand how this relationship differs by condition and across age groups.

Our findings also likely reflect patterns of reporting. Results for psychological violence closely mirror those for experience of any type of interpersonal violence, demonstrating that the “any interpersonal violence” model is driven predominantly by violence of this type. This is likely because the types of psychological violence included in the survey are by far the most commonly reported type of violence victimization, especially within the context of intimate partner violence [50]. Sexual violence, however, was the least commonly reported type of interpersonal violence, which may reflect norms that sexual violence often tends to be underreported [51, 52]. We found that highest level of educational attainment (college degree or higher) appeared to be positively associated with reporting psychological and sexual violence, which might reflect a higher likelihood to identify an experience as psychological violence or disclose experiencing psychological violence, rather than a higher likelihood of having such an experience. Alternatively, it may point to the presence of workplace or institutional forms of psychological violence that might affect individuals with greater educational exposure. Future research is needed to further investigate these patterns.

While quantitative intersectional approaches like MAIHDA are valuable for empirically understanding patterns of risk of violence victimization, qualitative and mixed-methods studies are also essential to understand how intersecting identities and socioeconomic factors may impact experiences of violence. Based on current understanding, intersectionality should be viewed less as the product of a specific analytic method and more as a framework to aid in making different populations and their complex identities more visible in research, how complex phenomena (violence in this case) are defined and measured, and how these findings are interpreted and put into practice, with an emphasis on recognizing power relations and structural drivers of inequality that may manifest in different populations in distinct ways [53]. 

Our results revealed meaningful differences in the predicted probability of experiencing interpersonal violence across intersectional strata, but we found that, in line with other applications of this method to the study of interpersonal violence, a relatively modest proportion of the difference in interpersonal violence reports was attributable to interactions, suggesting that risk of experiencing (or reporting) interpersonal violence in this study accumulates largely in additive rather than multiplicative ways. This pattern has been noted in other quantitative intersectional analyses using MAIHDA [54], and does not diminish the relevance of intersectionality in experience of interpersonal violence. Instead, it highlights that heterogeneity exists within intersectional strata. Additional structural or systemic factors (such as housing quality and stability, employment and occupation, neighborhood and built environment factors or stigma associated with gender identity, sexual orientation, or disability) that may not be captured by the variables included in this analysis may be drivers of this heterogeneity, providing additional individual-level experiences of structural and systemic violence that may intersect with those included here and shape vulnerability to and experience of interpersonal violence. However, a major contribution of this approach has been the identification of some of the most vulnerable population strata, pointing to the importance of intersectional targeting for public health and social policy interventions aimed to reduce or prevent violence victimization.

### Limitations

This study has several limitations. The cross-sectional nature of PNS data limits our ability to establish causality or observe temporal trends for all included variables. While reverse causality is primarily a concern for the relationship between experiencing any type of violence and health problems, our measure of long-term illness or disability was constructed to represent health problems that occurred more than one year prior to the report of violence and excluded mental health problems. The measures of violence used in this analysis are self-reported, which may be influenced by recall or social desirability bias, and underreporting is likely, especially of sexual violence. We note that the context of the interview may have affected the likelihood of reporting violence in some cases. Appendix Table 4 shows differences in the prevalence of most forms of violence when comparing those who were interviewed in a private versus a non-private location. While many of these differences are statistically significant, their differences are fairly are low in magnitude when compared to overall prevalence rates. For this reason, excluding those interviewed in a non-private location did not significantly affect results reported here. The PNS captures limited contextual information about the nature of violent experiences, limiting our ability to examine reported experience of violence in more detail. Additional factors such as neighborhood safety, family structure, or gender identity would be important to explore in future studies but were not examined here.

## Conclusion

Given the importance of structural factors and the way they help shape individual vulnerabilities, this analysis supports calls to consider interventions that directly tackle poverty and other aspects of social inequalities in Brazil. This could include greater eligibility for and/or increased benefits from Brazil’s conditional cash transfer program, *Bolsa Familia*, which has been found to be positively associated with decreasing some aspects of poverty and improving health outcomes [55]. To date, however, evidence on the effects of cash transfer programs on interpersonal violence have been somewhat mixed [56–58] and the protective effects of such programs may not accrue to all participants in equal measure [59]. So, while structural interventions are clearly needed to decrease poverty and other forms of violence, there remains an unmet need for better tailored individual-level interventions for survivors that include legal, mental health, housing assistance, child protective, and other social services [60]. 

Future research should incorporate different dimensions of socioeconomic status such as occupation, given differential exposure to violence in the workplace. Future work could also further enhance geographic context through linkage with contextual-level variables, such as those that describe neighborhood-level social, economic, and built environments, which may further shape underlying factors that shape violence victimization and resilience.

In conclusion, this study contributes novel evidence on the intersectional sociodemographic determinants of interpersonal violence in Brazil using a nationally representative dataset and robust quantitative methods. By illustrating the ways multiple dimensions of inequality (or structural violence) affect experiences of interpersonal violence, our findings highlight the need for comprehensive public health and social policy interventions to address both the structural drivers of violence—such as gender, racial, and wealth inequality—as well as interventions and ongoing support for high-risk populations. Results additionally have potential to inform programs and services that help those who have experienced violence to recover.

## Supplementary Information

Below is the link to the electronic supplementary material.

Supplementary Material 1

## Data Availability

The datasets analyzed during the current study are available from the Brazilian Institute of Geography and Statistics (IBGE) at the following URL: (https://www.ibge.gov.br/en/statistics/social/health/16840-national-survey-of-health.html?=&t=o-que-e).
