# Supplementary material for: Intersecting inequalities in experiences of violence among Brazilian adults: a multilevel analysis of individual heterogeneity and discriminatory accuracy (MAIHDA) of the 2019 National Health Survey
Source: Int J Equity Health. 2026 Mar 24;25:84. doi: 10.1186/s12939-026-02818-x (PMC13047797; doi:10.1186/s12939-026-02818-x)
Supplement: Supplementary file 1 — Supplementary Material 1 [file 12939_2026_2818_MOESM1_ESM.docx]

**Appendix Table 1: Full logistic regression analysis, by outcome**

|  | Psychological | physical | sexual | any | >1 |
| --- | --- | --- | --- | --- | --- |
| Male | 0.86*** | 0.94 | 0.43*** | 0.88*** | 0.78** |
| V female | 0.80,0.92 | 0.81,1.09 | 0.30,0.60 | 0.82,0.94 | 0.67,0.92 |
| 25-34 | 0.72*** | 0.72*** | 0.63* | 0.7*** | 0.71*** |
| V 18-24 | 0.64,0.82 | 0.61,0.86 | 0.43,0.91 | 0.62,0.79 | 0.59,0.85 |
| 35-44 | 0.66*** | 0.55*** | 0.4*** | 0.64*** | 0.52*** |
|  | 0.59,0.74 | 0.44,0.68 | 0.26,0.61 | 0.58,0.72 | 0.41,0.65 |
| 45-54 | 0.51*** | 0.4*** | 0.57 | 0.49*** | 0.46*** |
|  | 0.45,0.58 | 0.32,0.50 | 0.27,1.23 | 0.43,0.56 | 0.35,0.61 |
| 55-64 | 0.38*** | 0.26*** | 0.16*** | 0.37*** | 0.23*** |
|  | 0.33,0.44 | 0.20,0.35 | 0.09,0.29 | 0.33,0.43 | 0.17,0.31 |
| 65+ | 0.23*** | 0.13*** | 0.08*** | 0.22*** | 0.11*** |
|  | 0.19,0.27 | 0.09,0.18 | 0.04,0.15 | 0.19,0.25 | 0.08,0.16 |
| Black | 1.09*** | 1.3*** | 0.79 | 1.11*** | 1.13*** |
| V others | 1.01,1.18 | 1.13,1.48 | 0.52,1.20 | 1.03,1.20 | 1.00,1.34 |
| Single | 1.31*** | 1.6*** | 2.86*** | 1.33*** | 1.7*** |
|  | 1.21,1.41 | 1.34,1.92 | 1.85,4.42 | 1.24,1.43 | 1.38,2.10 |
| Primary education | 1.08 | 1.1 | 0.9 | 1.06 | 1.14 |
| V less | 0.97,1.20 | 0.90,1.34 | 0.51,1.59 | 0.96,1.18 | 0.91,1.42 |
| Secondary | 0.99 | 0.78* | 0.74 | 0.97 | 0.79 |
|  | 0.90,1.08 | 0.64,0.96 | 0.43,1.25 | 0.89,1.06 | 0.62,1.00 |
| Tertiary | 1.28*** | 0.74** | 0.98 | 1.24*** | 0.8 |
|  | 1.13,1.45 | 0.59,0.93 | 0.57,1.69 | 1.10,1.40 | 0.62,1.02 |
| Wealth tertile 2 | 0.97 | 0.81** | 1.32 | 0.97 | 0.85 |
| V 1 (poorest) | 0.89,1.05 | 0.70,0.94 | 0.79,2.21 | 0.89,1.05 | 0.71,1.03 |
| Wealth tertile 3 | 0.86** | 0.68*** | 0.84 | 0.85** | 0.68*** |
|  | 0.77,0.95 | 0.56,0.84 | 0.49,1.45 | 0.77,0.94 | 0.55,0.85 |
| Income quintile 2 | 0.93 | 0.99 | 0.59 | 0.93 | 0.91 |
|  | 0.84,1.03 | 0.83,1.17 | 0.34,1.04 | 0.84,1.03 | 0.74,1.11 |
| Income quintile 3 | 0.92 | 0.89 | 0.63 | 0.92 | 0.8 |
|  | 0.82,1.03 | 0.74,1.08 | 0.33,1.22 | 0.82,1.04 | 0.64,1.01 |
| Income quintile 4 | 0.95 | 0.98 | 0.79 | 0.95 | 0.95 |
|  | 0.82,1.10 | 0.74,1.30 | 0.39,1.59 | 0.83,1.10 | 0.69,1.32 |
| Income quintile 5 | 0.92 | 1 | 0.67 | 0.94 | 0.89 |
|  | 0.79,1.06 | 0.78,1.29 | 0.30,1.47 | 0.81,1.08 | 0.67,1.20 |
| Long-standing | 1.44*** | 1.46*** | 1.21 | 1.43*** | 1.5*** |
| Illness/disability | 1.34,1.56 | 1.26,1.69 | 0.78,1.90 | 1.32,1.54 | 1.26,1.79 |
| NE region | 1.13* | 1.04 | 1.14 | 1.11 | 1.06 |
| V N | 1.02,1.25 | 0.88,1.23 | 0.82,1.59 | 0.99,1.23 | 0.89,1.27 |
| SE region | 1.24* | 1.21 | 1.02 | 1.22* | 1.28* |
|  | 1.10,1.40 | 1.00,1.46 | 0.64,1.62 | 1.08,1.36 | 1.04,1.57 |
| S region | 1.12 | 1.23 | 0.65 | 1.11 | 1.2 |
|  | 0.98,1.28 | 0.98,1.55 | 0.38,1.11 | 0.97,1.26 | 0.94,1.53 |
| MW region | 1.07 | 1.05 | 1.15 | 1.06 | 1.09 |
|  | 0.94,1.21 | 0.84,1.30 | 0.75,1.77 | 0.94,1.20 | 0.87,1.36 |
| N | 83924 | 83924 | 83924 | 83924 | 83924 |

**Appendix Figure 1: Frequency of reporting violence, by type**

Weighted frequencies from PNS 2019.

**Appendix Table 2: Descriptive statistics**

|  | **Total**  N=83,942 | **No reported violence**  N=69,340 | **Experienced any violence**  N=14,602 | **Difference^1^** |
| --- | --- | --- | --- | --- |
| Female | 53.21 | 52.53 | 56.21 | 0.0000 |
|  | [52.58,53.83] | [51.85,53.22] | [54.74,57.67] |  |
| Male | 46.79 | 47.47 | 43.79 |  |
|  | [46.17,47.42] | [46.78,48.15] | [42.33,45.26] |  |
| Black | 11.59 | 11.28 | 12.95 | 0.0005 |
|  | [11.17,12.02] | [10.84,11.73] | [12.04,13.92] |  |
| All other races | 88.41 | 88.72 | 87.05 |  |
|  | [87.98,88.83] | [88.27,89.16] | [86.08,87.96] |  |
| 18-29 years of age | 22.14 | 19.86 | 32.37 | 0.0000 |
|  | [21.55,22.75] | [19.24,20.48] | [30.91,33.86] |  |
| 30+ years | 77.86 | 80.14 | 67.63 |  |
|  | [77.25,78.45] | [79.52,80.76] | [66.14,69.09] |  |
| <College education | 79.7 | 80.28 | 77.11 | 0.0000 |
|  | [78.97,80.41] | [79.54,80.99] | [75.55,78.59] |  |
| College+ | 20.3 | 19.72 | 22.89 |  |
|  | [19.59,21.03] | [19.01,20.46] | [21.41,24.45] |  |
| Assets (low) | 33.34 | 32.94 | 35.13 | 0.0002 |
|  | [32.66,34.02] | [32.24,33.64] | [33.67,36.62] |  |
| Assets (middle) | 33.33 | 33.09 | 34.39 |  |
|  | [32.67,34.00] | [32.36,33.83] | [33.00,35.81] |  |
| Assets (high) | 33.33 | 33.97 | 30.48 |  |
|  | [32.51,34.16] | [33.08,34.86] | [28.84,32.17] |  |
| Partnered | 43.75 | 45.79 | 34.59 | 0.0000 |
|  | [43.06,44.43] | [45.07,46.51] | [33.04,36.18] |  |
| Single | 56.25 | 54.21 | 65.41 |  |
|  | [55.57,56.94] | [53.49,54.93] | [63.82,66.96] |  |
| Long-term | 39.15 | 37.78 | 38.9 | 0.0874 |
| Illness/condition | [38.49,39.81] | [36.36, 39.23] | [38.29,39.51] |  |
| Urban residence | 86.59 | 85.93 | 89.54 | 0.0000 |
|  | [86.18,86.99] | [85.49,86.35] | [88.70,90.32] |  |
| Rural residence | 13.41 | 14.07 | 10.46 |  |
|  | [13.01,13.82] | [13.65,14.51] | [9.68,11.30] |  |

Numbers are weighted percentages and 95% confidence intervals from the PNS 2019

1. Difference in proportion of those not reporting and those reporting any interpersonal violence. P-value from Chi-Squared test with Rao-Scott correction.

**Appendix Table 3: Measures of experience of interpersonal violence included in the PNS 2019**

| **Type of Interpersonal Violence** | **PNS 2019 Survey Measure** |
| --- | --- |
| Psychological | In the **last twelve months**, has someone: Offended, humiliated, or ridiculed you in front of other people? |
| Psychological | In the last twelve months, has someone: Yelled at you or insulted you? |
| Psychological | In the last twelve months, has someone: Used social media or a cell phone to threaten, insult, swear at, or share images of you without your consent? |
| Psychological | In the last twelve months, has someone threatened to hurt or harm someone important to you? |
| Psychological | In the last twelve months, has someone: Deliberately destroyed something of yours? |
| Physical | In the last twelve months, has someone: Slapped or hit you? |
| Physical | In the last twelve months, has someone: Pushed you, held you tightly, or thrown something at you with the intention of hurting you? |
| Physical | In the last twelve months, has someone: punched you, kicked you, or dragged you by your hair? |
| Physical | In the last twelve months, has someone: Tried to or actually strangled, suffocated, or burned you on purpose? |
| Physical | In the last twelve months, has someone: Threatened or injured you with a knife, firearm, or some other weapon or object? |
| Sexual | In the last twelve months, has someone: touched, manipulated, kissed, or exposed parts of your body against your will? |
| Sexual | In the last twelve months, has anyone: Threatened you or forced you to have sexual relations or any other sexual acts against your will? |
| Sexual | And at some point in your **life**, has someone touched, manipulated, kissed, or exposed parts of your body against your will? |
| Sexual | Have you **ever** been threatened or forced to have sexual relations or any other sexual acts against your will? |

For all questions, response options include: Yes, No, and Not Applicable.

**Appendix Table 4: Weighted prevalence of each response by privacy status**

| Measure | Total | Not in a private location^1^ | In a private location^1^ | p-value^2^ |
| --- | --- | --- | --- | --- |
| Psychological | 17.4% | 13.1% | 17.8% | 0.000 |
| Physical | 4.2% | 3.2% | 4.3% | 0.017 |
| Sexual | 0.8% | 0.6% | 0.8% | 0.249 |
| Any type of violence | 18.3% | 13.9% | 18.7% | 0.000 |
| More than 1 type | 3.7% | 2.7% | 3.8% | 0.013 |

1. Prior to this section of the survey, interviewers were asked to note if the interviewee was in a private location.

2. P-value from Rao-Scott corrected chi-squared test of association between report of violence and report of being in a private location.
